# Terminology for “Elder Orphans” and Aging Well: Protocol for a Scoping Review

**DOI:** 10.2196/107175

**Published:** 2026-11-05

**Authors:** Gerald Yong Qi Tan, Emma Linh Shipalesky, Miho Asano

**Affiliations:** 1 Faculty of Health and Social Development, School of Health and Exercise Sciences, University of British Columbia, Okanagan Campus, 1147 Research Road, Kelowna, BC, V1V 1V7, Canada, 1 2508783517

**Keywords:** aging, aging in place, older adults, geriatrics, scoping review

## Abstract

**Background:**

Elder orphans have been described as a growing vulnerable population of isolated older adults with limited social and caregiving support. There is a lack of consistency in the terminology and definitions of elder orphans. This lack of consistency impedes research and implementation of services for elder orphans, who are predisposed to isolation, loneliness, unmet care needs, and a decline in health outcomes. It is imperative for elder orphans to plan for their health and well-being as they age in the community.

**Objective:**

The objectives of the scoping review are (1) to document the definitions and settings surrounding terms used to describe elder orphans, (2) to explore the determinants of isolation and loneliness in elder orphans, and (3) to understand the barriers to and facilitators of planning to age well in elder orphans.

**Methods:**

This review follows the JBI methodology for scoping reviews and the PRISMA-ScR (Preferred Reporting Items for Systematic Reviews and Meta-Analyses Extension for Scoping Reviews) guidelines. The search strategy was first developed and piloted in the Scopus database on October 16, 2025, to identify initial articles on the topic. The following databases were searched: Scopus, MEDLINE ALL (Ovid), CINAHL Complete (EBSCOhost), APA PsycINFO (EBSCOhost), Social Work Abstracts (EBSCOhost), Web of Science (Clarivate), Sociological Abstracts (ProQuest), Sociology Database (ProQuest), and Applied Social Sciences Index & Abstracts (ASSIA; ProQuest). Gray literature was searched using Google (incognito) and ClinicalTrials.gov. Only literature in English was considered due to staff constraints. Two reviewers will screen and extract data independently using piloted screening and data extraction tools. Descriptive analysis will include narrative summaries, tables, and charts and deductive thematic analysis using appropriate frameworks.

**Results:**

This protocol has been registered on the Open Science Framework. As of October 2026, the final search and abstract citation screening has been completed. We expect screening, data extraction, and synthesis to be completed by February 2027, with the manuscript of the scoping review submitted thereafter.

**Conclusions:**

This review explores both peer-reviewed and gray literature using rigorous and reproducible methods. It will support researchers in streamlining future work on elder orphans and will identify directions for future research and services for elder orphans.

## Introduction

### Background

Elder orphans have been defined as “aged, community-dwelling individuals who are socially and/or physically isolated, without an available known family member or designated surrogate or caregiver” [1]. A US study analyzing national survey data from 6680 community-dwelling older adults estimated that about 3% were elder orphans [2]. The same study estimated that an additional 21% of those older adults were at risk. To our knowledge, this has been the only study to estimate the prevalence of elder orphans to date. Some other common terms used to describe this population are “older adults without advocates” and “solo-agers” [1,3]. Researchers and clinicians have expressed issues with the use of the term “elder orphan,” for example, suggesting it promotes stigmatization, especially when used in lay discourse [3,4]. In contrast, empirical evidence suggests that elder orphans self-identified with this term as it may raise awareness to health and social care service providers [5]. This review will use the term “elder orphans” to align with the definition provided by Carney and colleagues [1]. Their seminal work remains most cited within the population of elder orphans (including alternative terminology), making “elder orphans” a useful operational term for our review [3,6,7]. Previous work by others has recommended targeting social isolation and loneliness as priority areas for improving the health and well-being of elder orphans [8,9]. As summarized in a recent review, isolation is typically defined as an objective, social concept of having limited social interactions, while loneliness is a subjective concept of the dissatisfaction with one’s social connections [10]. Little is known about the determinants of isolation and loneliness in elder orphans [8]. Both isolation and loneliness are associated with declines in mental and physical health and increased mortality [11]. Based on 2 systematic reviews, the global prevalence of isolation and loneliness among older adults is 33% and 28%, respectively [12,13]. Another identified health and well-being priority for elder orphans is to prevent them from becoming unrepresented, a state where one lacks decision-making capacity, advance directives (legal documentation for health care decision-making preferences), and advocates [8,14]. Health care decision-making is complex for unrepresented patients and incurs significant, avoidable costs [15,16]. The process of planning for and documenting advance directives, also known as advance care planning (ACP), has therefore been a priority area of research on elder orphans [8,16].

### Rationale

Research on elder orphans is emerging, but there is still no consensus on appropriate terminology [3,5-7]. Consistency in terminology is crucial to facilitate identification of elder orphans and for resource allocation in research and services for elder orphans [1,6]. Beyond medical and health care settings, our understanding of planning to age well while remaining in a community setting is limited [8]. Based on a systematic review, aging is a multifaceted process, and planning to age well includes not only health but also psychological, financial, housing, social, and active leisure planning [17]. Little is known about planning to age well for elder orphans. In our review, planning to age well will include ACP and other forms of planning related to living arrangements, finances, and concepts linked to maintaining the psychosocial well-being of elder orphans while living in the community.

### Objectives

A scoping review is ideal as it can “clarify key concepts/definitions” and “identify and analyze knowledge gaps,” ultimately achieving our review objectives [18]. The objectives of this scoping review are (1) to document the definitions and settings of terms used to describe elder orphans, (2) to explore the determinants of isolation and loneliness in elder orphans, and (3) to understand the barriers to and facilitators for planning to age well in elder orphans. This review will support researchers in streamlining future work on elder orphans and identifying directions for future research. We operationalized “elder orphans” as aged 50 and above to comprehensively document elder orphan terminology according to our first objective. We have selected this age range as it has been used by major organizations on aging support such as the AARP (United States) and the National Institute on Aging (Canada) [19,20].

## Methods

### Design and Guidelines

The review will be guided by the JBI methodology for scoping reviews evidence synthesis [21]. The results will be reported according to PRISMA-ScR (Preferred Reporting Items for Systematic Reviews and Meta-Analyses Extension for Scoping Reviews) guidelines [22]. This protocol was developed in alignment with the PRISMA-P (Preferred Reporting Items for Systematic Reviews and Meta-Analyses Protocols) extension [23], and a checklist is documented in Checklist 1 [23]. This protocol has been registered on the Open Science Framework (OSF) [24].

### Eligibility Criteria

The eligibility criteria were defined in advance based on the research questions and the population, concept, and context framework as recommended by the JBI. In summary, the outcomes of interest are (1) elder orphan terminology and definitions, (2) determinants of isolation and loneliness, and (3) barriers to and facilitators of planning to age well.

#### Population

The review includes literature exploring older adults aged 50 and above living alone in the community with at least 1 additional characteristic of an elder orphan. Characteristics include having no (1) available family member, (2) surrogate/advocate, or (3) caregiver. Literature on the general population of older adults will be excluded. Literature with less than 50% of the sample being elder orphans will be excluded.

#### Concept

We will include literature that uses terms such as “elder orphans,” “adult orphans,” “older adults without advocates,” “unrepresented older adults” and “solo-agers.” These are some of the terms used in the literature to describe elder orphans, which we identified in our pilot searches.

Literature that explores either the determinants of isolation and/or loneliness in elder orphans or the barriers to and facilitators of planning to age well in elder orphans will be included. Isolation refers to having limited interactions with others, including a lack of engagement in social activities, and loneliness refers to a feeling of dissatisfaction with one’s social connections [10]. Planning to age well, as defined in the Introduction, includes ACP and other forms of planning related to the well-being of elder orphans while remaining in the community.

#### Context

We will include literature on independent community dwelling. Literature based in other settings only, such as in-patient or long-term care settings, will be excluded.

### Types of Sources

Sources include peer-reviewed literature using any study design, that is, qualitative, quantitative, and mixed methods studies and reviews, published in indexed literature. Gray literature, such as dissertations, theses, preprints, guidelines, reports, and blogs, will be considered for inclusion if the eligibility criteria are met. Up to 2 attempts will be made to contact authors to request for missing or additional data, if necessary. We will only consider literature published in English as translation accuracy cannot be verified due to staff limitations. Forward and backward citation search will be conducted for all relevant literature.

### Search Strategy

The search strategy was formulated by the research team, with the support of 2 librarians with backgrounds in social sciences and health sciences, respectively. Given the limited evidence available on elder orphans [8], the search strategy aims to uncover all literature related to elder orphans, hence we did not apply concept-specific terminology limiters of isolation, loneliness, and planning. The strategy was first developed and piloted in the Scopus database on October 16, 2025, to identify initial articles on the topic (Multimedia Appendix 1). Titles, abstracts, and index terms were screened to determine relevant search terms for the complete search strategy. We found that index terms were not consistently used among relevant articles. Further, very limited articles used index terms specific to our population (eg, “elder orphan” is only indexed for 2 articles and “adult orphan” is not indexed at all). After discussion with the librarians, we decided that the complete search strategy would exclude index terms. Our search strategy targets both peer-reviewed and gray literature, using a 3-step approach similar to previous systematic and scoping reviews that include gray literature [25,26]:

- Step 1: peer-reviewed literature database search
- Step 2: Google search engine web search using incognito mode and ClinicalTrials.gov search
- Step 3: forward and backward citation search for all relevant articles identified above

We refined and reconducted the search in Scopus and adapted it for the following databases on November 3, 2025: Scopus, MEDLINE ALL (Ovid), CINAHL Complete (EBSCOhost), APA PsycINFO (EBSCOhost), Social Work Abstracts (EBSCOhost), Web of Science (Clarivate), Sociological Abstracts (ProQuest), Sociology Database (ProQuest), and Applied Social Sciences Index & Abstracts (ASSIA; ProQuest). We found no new terms, and the final searches were conducted on May 29, 2026, in all databases and on Google (Multimedia Appendix 2). We added the database ClinicalTrials.gov and conducted a search on October 15, 2026. Our searches applied Boolean operators (“AND” and “OR”), proximity operators (eg, W/5), and truncations (*), depending on the databases’ specifications, in all fields. Only literature published since 1993 were considered as the term “elder(ly) orphan” was first introduced then [1].

Gray literature was searched for via ClinicalTrials.gov and Google web search (first 200 results) using incognito mode to depersonalize results, as described by Brennan and colleagues [25]. For the Google web search, we used the limit of first 200 results, as compared to the norm of the first 100 [25,26], since our search continued to reveal relevant and unique articles. As elder orphans are a very specific and emerging population, we could not identify any specific gray literature databases, organizations, or websites targeting elder orphans. After discussion with the 2 librarians, we agreed that we would not use targeted website searches or gray literature databases. Literature from government, health, and social work organizations, as well as blog articles, were considered if they met the eligibility criteria.

### Study Selection and Data Extraction

#### Collation

Following the search, peer-reviewed and gray literature citations were collated and uploaded into Zotero (version 7.0.32; Corporation for Digital Scholarship and Roy Rosenzweig Center for History and New Media). These citations were uploaded to the systematic review software, Covidence (Veritas Health Innovation) [27], and were software-deduplicated for the subsequent screening process. Manual deduplication will subsequently be conducted by reviewers, if needed.

#### Screening

Titles and abstracts were first screened independently by 2 reviewers using a draft screening tool created according to the eligibility criteria. During this stage, 5% of the citations were randomly selected to pilot-test our screening tool. The reviewers independently screened these citations to ensure that the exclusion criteria can be followed consistently. The final screening tool can be found in Multimedia Appendix 3. Subsequently, relevant citations will have their full text screened by the same reviewers. Any changes to the screening tool at the full-text stage will be documented in the scoping review publication. Disagreements at both screening stages will be handled via discussion between the reviewers until consensus is reached. If consensus cannot be reached, another reviewer will be consulted. Reasons for exclusion at the full-text stage will be recorded and reported in a PRISMA (Preferred Reporting Items for Systematic Reviews and Meta-Analyses) flow diagram [22]. The results of the search and screening process will be reported in the results and documented in a PRISMA flow diagram.

#### Data Extraction

Data will be extracted by a minimum of 2 reviewers using a draft extraction tool created based on our objectives (Multimedia Appendix 4). During this stage, 5% of the citations will be randomly selected for pilot extraction. The reviewers will independently extract data from these citations to ensure that the tool can be used reliably by the reviewers. Any modifications to the tool will be documented in the scoping review. We will discuss any disagreements on data extracted. If consensus cannot be reached, another reviewer will be consulted. We will extract the following metadata: authors, date published, journal/website, study design, and country/region. We will also include participant demographics (eg, age, gender, and ethnicity) and details related to the population, concept, and context of interest. These include elder orphan terminology and definitions, determinants of isolation and loneliness, and barriers and facilitators in planning to age well.

#### Data Analysis and Presentation

Data will be organized according to each research question. Our first research question on definitions and settings of elder orphans will be addressed narratively and in a table and/or flowchart to represent the elements that make up each definition of elder orphans (eg, isolated, without family, without caregiver, or without advocate) and its settings. The second research question on the determinants of isolation and loneliness will be addressed using deductive thematic analysis according to a version of the social-ecological model [28-30], which has been endorsed by the World Health Organization [28]. This model has been applied to a scoping review investigating a similar topic, the broader population of older adults [31]. The third research question on planning to age well will be addressed using deductive thematic analysis according to the 6 dimensions of preparation for aging, as identified in a systematic review by Solhi and colleagues [17]. We will follow the 5 steps of deductive qualitative analysis as recommended by Fife and Gossner [32]. The initial steps, (1) developing the research question and selecting guiding theory and (2) operationalizing theory, have been conducted during the development of this protocol. To operationalize theory, themes were defined based on the 4 levels of the social-ecological model and the 6 dimensions of preparation for aging, respectively. The next step, (3) gathering a purposive sample, has been modified to fit the context of a scoping review, and hence the sample will be the articles that are screened in. The final steps, (4) coding and analyzing data and (5) theorizing, will be conducted by at least 2 reviewers to enhance reliability and reduce researcher bias during interpretation. Metadata and demographic information will be presented in a tabular format. A geographical map or timeline may be used if noteworthy trends emerge from the country of origin or date published, respectively. Other frameworks will be considered if they are more appropriate to present our data.

## Results

This protocol has been registered on OSF [24]. As of October 2026, title and abstract citation screening (n=1882) has been completed, and full-text screening (n=505) is underway. We expect screening, data extraction, and synthesis to be completed by February 2027, with the manuscript of the scoping review submitted thereafter.

## Discussion

### Principal Results

Our scoping review is expected to comprehensively document elder orphan terminology, their definitions, and their settings. We anticipate that there will be variations in the way elder orphans are described. These may include different terms to describe the same population and/or the same terms being used to describe different populations. We expect that our review will also synthesize knowledge surrounding isolation and loneliness and planning to age well for elder orphans. We anticipate limited literature surrounding these concepts of aging well as an elder orphan and instead a greater focus on the health care and medical aspects of aging well, such as physical health and ACP. Across our 3 research objectives, we predict that there will be substantial knowledge contributions from gray literature, which was not considered in previous work by others.

### Limitations

Our review considers only English-language publications, which may exclude relevant international articles. As with most scoping reviews, we will not conduct formal critical appraisal or risk-of-bias assessment.

### Comparison With Prior Work

Previous studies on elder orphans have recommended the use of consistent terminology, and identified isolation, loneliness, and planning to age well as some of the key research directions for elder orphans. These recommendations have been incorporated into our review objectives. While some reviews have been conducted on elder orphans, our review further uses a rigorous and comprehensive search, such as including gray literature. Prior works have focused on the health outcomes of elder orphans and planning in the health care context (eg, ACP). Our work focuses on the determinants of isolation and loneliness rather than its health outcomes. We also consider other aspects of planning and use a framework to consolidate planning into themes of health, psychological, financial, housing, social, and active leisure planning.

### Conclusions

This protocol documents a systematic and comprehensive peer-reviewed and gray literature scoping review. Where possible, we have used means to enhance rigor and transparency. It will support researchers in streamlining future work on elder orphans and will identify directions for future research. It may also contribute to service planning and implementation for elder orphans.

## Supplementary material

10.2196/107175 Multimedia Appendix 1 Pilot search strategy.

10.2196/107175 Multimedia Appendix 2 Full search strategy.

10.2196/107175 Multimedia Appendix 3 Screening tool.

10.2196/107175 Multimedia Appendix 4 Draft extraction tool.

10.2196/107175 Checklist 1 PRISMA-P checklist.

## Abbreviations

ACP: advance care planning
ASSIA: Applied Social Sciences Index & Abstracts
OSF: Open Science Framework
PRISMA: Preferred Reporting Items for Systematic Reviews and Meta-Analyses
PRISMA-P: Preferred Reporting Items for Systematic Reviews and Meta-Analyses Protocols
PRISMA-ScR: Preferred Reporting Items for Systematic Reviews and Meta-Analyses Extension for Scoping Reviews
