# Supplementary material for: Terminology for “Elder Orphans” and Aging Well: Protocol for a Scoping Review
Source: JMIR Res Protoc. 2026 Nov 5;15:e107175. doi: 10.2196/107175 (PMC13739265; doi:10.2196/107175)
Supplement: Supplementary file 1 [file resprot-v15-e107175-s001.docx]

Multimedia Appendix 1: Pilot search strategy

| Scopus | |
| --- | --- |
| Search Conducted: October 16, 2025 | |
| Search | Query (Records Retrieved) |
| 1 | elder* orphan? (133) |
| 2 | adult orphan? (67) |
| 3 | "ag*ing solo" OR "solo ag*ing" OR "solo-ag*ing" (40) |
| 4 | "solo ager*" OR "solo-ager*" (34) |
| 5 | ag*ing alone (65) |
| 6* | "living alone" OR "live* alone" (11173) |
| 7 | Unrepresented (255) |
| 8 | Kinless (25) |
| 9 | Surrogateless (2) |
| 10 | Unbefriended (194) |
| 11* | "isolat*" (448835) |
| 12 | ("no kin" OR "without kin") W/5 (adult? OR patient? OR elder*) (3) |
| 13 | "no surrogate?" OR "without surrogate?" W/5 (adult? OR patient? OR elder*) (187) |
| 14 | "no caregiv*" OR "without caregiv*" W/5 (adult? OR patient? OR elder*) (77) |
| 15 | "no care partner?" OR "without care partner?" W/5 (adult? OR patient? OR elder*) (2) |
| 16 | "no advocate?" OR "without advocate?" W/5 (adult? OR patient? OR elder*) (22) |
| 17* | ( ( "elder* orphan" ) OR ( "adult orphan" ) OR ( "ag*ing solo" OR "solo ag*ing" OR "solo-ag*ing" ) OR ( "solo ager" OR "solo-ager" ) ) OR ( ( "ag*ing alone" OR "unrepresented" OR "kinless" OR "surrogateless" OR "unbefriended" OR "no kin" OR "without kin" OR "no surrogate" OR "without surrogate" OR "no caregiv*" OR "without caregiv*" OR "no care partner" OR "without care partner" OR "no advocate" OR "without advocate" ) W/5 ( adult OR patient OR elder* ) ) (816) |
| Published in English, from 1993.  *Search #6 and #11 were not used in the combined search (#17) as they yielded too many irrelevant results. Search #17 was rerun in Scopus and adapted in other databases for the full search | |
