# Supplementary material for: Terminology for “Elder Orphans” and Aging Well: Protocol for a Scoping Review
Source: JMIR Res Protoc. 2026 Nov 5;15:e107175. doi: 10.2196/107175 (PMC13739265; doi:10.2196/107175)
Supplement: Supplementary file 2 [file resprot-v15-e107175-s002.docx]

Multimedia Appendix 2: Full search strategy

| Search Conducted: May 29, 2026; Oct 15, 2026 (ClinicalTrials.gov) | |
| --- | --- |
| Database (Platform) | Query [Records Retrieved] |
| PsycINFO (EBSCOhost) | ( ( "elder* orphan#" ) OR ( "adult orphan#" ) OR ( "ag#ing solo" OR "solo ag#ing" OR "solo-ag#ing") OR ( "solo ager#" OR "solo-ager#" ) ) OR ( ( "ag#ing alone" OR "unrepresented" OR "kinless" OR "surrogateless" OR "unbefriended" OR "no kin" OR "without kin" OR "no surrogate#" OR "without surrogate#" OR "no caregiv*" OR "without caregiv*" OR "no care partner#" OR "without care partner#" OR "no advocate#" OR "without advocate#" ) N5 ( adult# OR patient# OR elder* ) ) [177] |
| CINAHL Complete (EBSCOhost) | ( ( "elder* orphan#" ) OR ( "adult orphan#" ) OR ( "ag#ing solo" OR "solo ag#ing" OR "solo-ag#ing") OR ( "solo ager#" OR "solo-ager#" ) ) OR ( ( "ag#ing alone" OR "unrepresented" OR "kinless" OR "surrogateless" OR "unbefriended" OR "no kin" OR "without kin" OR "no surrogate#" OR "without surrogate#" OR "no caregiv*" OR "without caregiv*" OR "no care partner#" OR "without care partner#" OR "no advocate#" OR "without advocate#" ) N5 ( adult# OR patient# OR elder* ) ) [90] |
| Social Work Abstracts (EBSCOhost) | ( ( "elder* orphan#" ) OR ( "adult orphan#" ) OR ( "ag#ing solo" OR "solo ag#ing" OR "solo-ag#ing") OR ( "solo ager#" OR "solo-ager#" ) ) OR ( ( "ag#ing alone" OR "unrepresented" OR "kinless" OR "surrogateless" OR "unbefriended" OR "no kin" OR "without kin" OR "no surrogate#" OR "without surrogate#" OR "no caregiv*" OR "without caregiv*" OR "no care partner#" OR "without care partner#" OR "no advocate#" OR "without advocate#" ) N5 ( adult# OR patient# OR elder* ) ) [3] |
| Sociological Abstracts (ProQuest) | "elder* orphan?" or "adult orphan?" or "ag?ing solo" or ("solo ag?ing" or "solo-ag?ing") or ("solo ager?" or "solo-ager?") or (("ag?ing alone" or "unrepresented" or "kinless" or "surrogateless" or "unbefriended" or "no kin" or "without kin" or "no surrogate?" or "without surrogate?" or "no caregiv*" or "without caregiv*" or "no care partner?" or "without care partner?" or "no advocate?" or "without advocate?") NEAR/5 (adult? or patient? or elder*)) [109] |
| Sociology database (ProQuest) | "elder* orphan?" or "adult orphan?" or "ag?ing solo" or ("solo ag?ing" or "solo-ag?ing") or ("solo ager?" or "solo-ager?") or (("ag?ing alone" or "unrepresented" or "kinless" or "surrogateless" or "unbefriended" or "no kin" or "without kin" or "no surrogate?" or "without surrogate?" or "no caregiv*" or "without caregiv*" or "no care partner?" or "without care partner?" or "no advocate?" or "without advocate?") NEAR/5 (adult? or patient? or elder*)) [172] |
| Applied Social Sciences Index and Abstracts (ProQuest) | "elder* orphan?" or "adult orphan?" or "ag?ing solo" or ("solo ag?ing" or "solo-ag?ing") or ("solo ager?" or "solo-ager?") or (("ag?ing alone" or "unrepresented" or "kinless" or "surrogateless" or "unbefriended" or "no kin" or "without kin" or "no surrogate?" or "without surrogate?" or "no caregiv*" or "without caregiv*" or "no care partner?" or "without care partner?" or "no advocate?" or "without advocate?") NEAR/5 (adult? or patient? or elder*))  [106] |
| Scopus (Scopus) | ( "elder* orphan*" OR "adult orphan*" OR "ag*ing solo" OR "solo ag*ing" OR "solo-ag*ing" OR "solo ager*" OR "solo-ager*" ) OR ( ( "ag*ing alone" OR "unrepresented" OR "kinless" OR "surrogateless" OR "unbefriended" OR "no kin" OR "without kin" OR "no surrogate*" OR "without surrogate*" OR "no caregiv*" OR "without caregiv*" OR "no care partner*" OR "without care partner*" OR "no advocate*" OR "without advocate*" ) W/5 ( adult* OR patient* OR elder* ) ) [910] |
| Web of Science (Clarivate) | "elder* orphan$" or "adult orphan$" or "ag$ing solo" or ("solo ag$ing" or "solo-ag$ing") or ("solo ager$" or "solo-ager$") or (("ag$ing alone" or "unrepresented" or "kinless" or "surrogateless" or "unbefriended" or "no kin" or "without kin" or "no surrogate$" or "without surrogate$" or "no caregiv*" or "without caregiv*" or "no care partner$" or "without care partner$" or "no advocate$" or "without advocate$") and (adult$ or patient$ or elder$)) [797] |
| Medline (Ovid) | "elder* orphan?" or "adult orphan?" or "ag?ing solo" or ("solo ag?ing" or "solo-ag?ing") or ("solo ager?" or "solo-ager?") or (("ag?ing alone" or "unrepresented" or "kinless" or "surrogateless" or "unbefriended" or "no kin" or "without kin" or "no surrogate?" or "without surrogate?" or "no caregiv*" or "without caregiv*" or "no care partner?" or "without care partner?" or "no advocate?" or "without advocate?") adj5 (adult? or patient? or elder*)) [201] |
| Google Web (Search Engine) | "elder* orphan*" OR "adult* orphan*" OR "ag*ing solo" OR "solo ag*ing" OR "solo-ag*ing" OR "solo ager*" OR "solo-ager*" OR "ag*ing alone" OR "unrepresented adult*" OR "unrepresented elder*" OR "unrepresented patient*" OR "kinless adult*" OR "kinless elder*" OR "kinless patient*" OR "surrogateless adult*" OR "surrogateless elder*" OR "surrogateless patient*" OR "unbefriended adult*" OR "unbefriended elder*" OR "unbefriended patient*" OR "no caregiv*" AROUND(5) adult* OR "no caregiv*" AROUND(5) elder* OR "no caregiv*" AROUND(5) patient* OR "no surrogate*" AROUND(5) adult* OR "no surrogate*" AROUND(5) elder* OR "no surrogate*" AROUND(5) patient* OR "no advocate*" AROUND(5) adult* OR "no advocate*" AROUND(5) elder* OR "no advocate*" AROUND(5) patient* OR "without caregiv*" AROUND(5) adult* OR "without caregiv*" AROUND(5) elder* OR "without caregiv*" AROUND(5) patient* OR "without surrogate*" AROUND(5) adult* OR "without surrogate*" AROUND(5) elder* OR "without surrogate*" AROUND(5) patient* OR "without advocate*" AROUND(5) adult* OR "without advocate*" AROUND(5) elder* OR "without advocate*" AROUND(5) patient* OR "no care partner*" AROUND(5) adult* OR "no care partner*" AROUND(5) elder* OR "no care partner*" AROUND(5) patient* OR "without care partner*" AROUND(5) adult* OR "without care partner*" AROUND(5) elder* OR "without care partner*" AROUND(5) patient* [200] |
| ClinicalTrials.gov | ( "elder orphan" OR "elder orphans" OR "elderly orphan" OR "elderly orphans" OR "adult orphan" OR "adult orphans" OR "aging solo" OR "solo aging" OR "solo-aging" OR "solo ager" OR "solo agers" OR "solo-ager" OR "solo-agers" ) OR ( ("aging alone" OR unrepresented OR kinless OR surrogateless OR unbefriended OR "no kin" OR "without kin" OR "no surrogate" OR "no surrogates" OR "without surrogate" OR "without surrogates" OR "no caregiver" OR "no caregivers" OR "without caregiver" OR "without caregivers" OR "no care partner" OR "no care partners" OR "without care partner" OR "without care partners" OR "no advocate" OR "no advocates" OR "without advocate" OR "without advocates") AND (adult OR adults OR patient OR patients OR elder OR elders OR elderly) ) [32] |
| **Total** | **2797** |
| Published in English, from 1993 (except ClinicalTrials.gov, which was from 2000 [its year of launch]). | |
