# Supplementary material for: Terminology for “Elder Orphans” and Aging Well: Protocol for a Scoping Review
Source: JMIR Res Protoc. 2026 Nov 5;15:e107175. doi: 10.2196/107175 (PMC13739265; doi:10.2196/107175)
Supplement: Supplementary file 3 [file resprot-v15-e107175-s003.docx]

Multimedia Appendix 3: Screening Tool

1. Is the population older adults living alone?

2. Does the citation describe psychosocial well-being?

This excludes citations focusing on biomedical outcomes of health such as biomarkers and physiological measures.

3. Are the older adults living in the community?

This includes older adults living in independent living facilities but excludes long-term care facilities (e.g. nursing homes) and hospitals.

4. Is the citation a post-publication disclosure, communication or correction?

This refers to corrections/errata, notes, letters or retractions.
