# Supplementary material for: Terminology for “Elder Orphans” and Aging Well: Protocol for a Scoping Review
Source: JMIR Res Protoc. 2026 Nov 5;15:e107175. doi: 10.2196/107175 (PMC13739265; doi:10.2196/107175)
Supplement: Supplementary file 4 [file resprot-v15-e107175-s004.docx]

Multimedia Appendix 4: Draft Data Extraction Tool

| Details | Source 1 | Source 2 | Source 3 |
| --- | --- | --- | --- |
| Author(s) |  |  |  |
| Date published |  |  |  |
| Title |  |  |  |
| Journal/volume/issue/page or website |  |  |  |
| Country of origin |  |  |  |
| Type of literature (Peer-reviewed or Grey) |  |  |  |
| Study design (If applicable) |  |  |  |
| Age of participants |  |  |  |
| Gender of participants |  |  |  |
| Ethnicity of participants |  |  |  |
| What was/were the term(s) used to describe elder orphans (EO)? |  |  |  |
| How was/were these term(s) defined? |  |  |  |
| In what setting(s) was/were these term(s) used? (e.g. community, long-term care facility) |  |  |  |
| What was/were the determinant(s) of loneliness in EO identified? |  |  |  |
| What was/were the determinant(s) of isolation in EO identified? |  |  |  |
| What was/were the barrier(s) to planning in EO identified? |  |  |  |
| What was/were the facilitator(s) to planning in EO identified? |  |  |  |
| EO, Elder orphans | | | |
